# A comparative analysis of diversification of agricultural production among EU candidate countries and Eastern Partnership countries: An international perspective

**DOI:** 10.1371/journal.pone.0314471

**Published:** 2025-03-03

**Authors:** Anna Jankowska, Tomáš Hlavsa

**Affiliations:** 1 Department of Economics and Economic Policy in Agribusiness, Faculty of Economics, Poznan University of Life Sciences, Poznan, Poland; 2 Department of Statistics, Faculty of Economics and Management, Czech University of Life Sciences, Prague, Czech Republic; Canakkale Onsekiz Mart University, TÜRKIYE

## Abstract

The volume and structure of agricultural production depend on many different factors, including environmental, climatic, weather, economic, development potential of the agricultural sector, but also on historical conditions, etc. This study aimed to determine the diversification of the obtained agricultural production per capita of the EU candidate countries (CC) and the Eastern Partnership countries (EPC), presenting the potential of the analysed countries in terms of selected products in the field of plant and animal production, as well as to present quantitative changes in production in the years 2011–2021. Food self-sufficiency indicators, cluster analysis using Ward’s method, a synthetic measure of agricultural production development and a composite indicator were applied in the study, to show the diversity and position of the surveyed countries in terms of agricultural production per capita. The research revealed that Belarus and Türkiye have the highest agricultural production potential. Belarus has the potential to be competitive on the market mainly of animal products. Three countries dominate the grain market: Moldova, Serbia and Ukraine. Countries where the agricultural sector does not play a significant role are primarily Bosnia and Herzegovina, Georgia and Montenegro.

## Introduction

One of the central elements of the development of each country’s economy is efficient and modern agriculture. The word ‘agriculture’ is derived from the Latin words *ager*, which means field, and *colo*, which means cultivate. Cultivation is the process by which people control and observe the life cycles of various plants. Agriculture is a science or practice of farming, cultivation of the soil for the growing of crops and the rearing of animals to provide food, fibre and other products for human consumption and use. It is a fundamental and essential aspect of human civilization, providing sustenance and raw materials for various industries.

The process of food production has changed significantly over time and over space. Such changes have been influenced by the dynamic interactions between improved technologies and increasing human population [1]. The evolving organisation and structure of agricultural production remain subjects of considerable interest [2,3]. It is worth emphasizing that agricultural production plays a very important role in the economy. Agriculture plays a critical role in ensuring food security by producing the necessary crops and livestock to feed the world’s population. It provides a reliable supply of nutritious food, essential for human survival and well-being. Secondly, agriculture is a primary source of employment and income for billions of people worldwide, particularly in rural areas. It supports the livelihoods of farmers, farm labourers, and individuals engaged in agribusiness, contributing to economic growth and poverty reduction. Agriculture contributes significantly to national economies by generating export earnings, promoting trade, and fostering economic development. It provides the foundation for related industries, such as food processing, manufacturing of agricultural machinery, and agrochemicals, creating employment and income opportunities across the value chain. Agricultural development often leads to improved rural infrastructure, including irrigation systems, roads, and storage facilities. These developments enhance access to markets, healthcare, education, and other social services, contributing to better living conditions and reducing rural-urban migration.

There are various methods and indicators for measuring agricultural production that are used around the world and are influenced by socio-economic characteristics, farmers’ characteristics and natural factors [4,5] and increasing efficiency and production is a key objective of the development of agricultural production.

So far, the research on agricultural production of CC and EPC has mainly focused on analysis of one product of agricultural production in one country. Corobov [6] analyzed, for example, crop production in Moldova, Ozkan et al. [7] citrus production in Türkiye, Avetisyan [8] production in Armenia, Karić et al. [9] vegetable production in Bosnia and Herzegovina, Melkonyan [10] crop production in Armenia, Zurovec et al. [11] production in Bosnia and Herzegovina, Glavić et al. [12] milk production in Bosnia and Herzegovina, Van Berkum [13] production in Azerbaijan, Cetin and Vardar [14] tomato production in Türkiye, Ciaian et al. [15] production in Albania, Bojkovska et al. [16] raspberry production in North Macedonia and Cvijanović et al. [17] production of niche products in Serbia. Other authors, in turn, conducted comparative analyses of only a few countries studied by the authors. For example, Kožar et al. [18] studied Armenia, Azerbaijan, Moldova, Ukraine, Russia, Georgia, Belarus and Kazakhstan; Millns [19] studied Armenia, Georgia and Moldova; Zhemoyda and Gerasymenko [20] studied Belarus, Moldova and Ukraine, among other countries. Those studies were mainly based on the document analysis, descriptive statistics, comparison and synthesis methods applied.

A few studies have used other research methods. For instance, Kollias and Messis [21] and Borys, Polgár & Zlate [22] used the convergence index, Brankov and Matkovski [23] analised the self-sufficiency index for chosen Balkan countries to investigate what factors influence the self-sufficiency of agricultural production in these countries, Potopova, Boroneanţ, Boincean and Soukup [24] used the non-parametric, Mann–Kendall and Pettitt tests, Hrybau, Hryshanava, Witkowska-Dąbrowska, Świdyńska [25] used selected per capita intensity indices, the change rate index and a logistic function, Dinçer [26] incorporated the Topsis method, Başarır, Karli and Bilgiç [27] used Cobb-Douglas production function and ridge regression estimating technique, Ilić, Jovanović and Janković–Milić [28] forecast production in Serbia using ARIMA model and Zhemoyda & Gerasymenko [20] used the following group of indicators of regional specialization and geographic concentration: Herfindahl index, dissimilarity indexes of specialization and concentration, Krugman specialization and dissimilarity indexes, and the GINI coefficient of regional specialization.

Nevertheless, there are not too many studies that show the diversification of agricultural production of all the CC and the EPC analysed together. Thus this paper analyses a currently important topic – the diversification of agricultural production of the EU candidate and Eastern Partnership countries.

This paper adds value to the existing literature mainly by providing an international approach to agricultural production, using several different statistical methods. In this respect, the approach proposed in this paper differs from other analyses cited above. Therefore, an important advantage of this study is a synthetic approach to the production of the agricultural sector issue, enabled by the use of a synthetic methods which take production into account, and provide a basis for the typological classification of the CC and the EPC.

The study of the diversification of the level of production using statistical methods is an important element in testing economic theories in the field of competitiveness research in the CC and EPC and it shows which countries may become competitive in the future in the EU market. Showing the importance of the diversification of the production development in the CC and the EPC we can observe its impact on the future integration process of the CC and EPC. The results of the research are important from the point of view of the contribution to the economy of European integration, both in theoretical and empirical terms, but above all in the field of sectoral policy, which is the Common Agricultural Policy (CAP). A comprehensive approach to the subject of research and its multidirectional nature, as well as the obtained results will be important both for the agricultural policy of the studied countries, as well as for the CAP.

As far as the limitations of the study are concerned the author is aware that because of the approach used and of its international nature, this study fails to take into account a number of major aspects of the development of production, such as for instance the historical or the institutional country-level variation in the aspects considered. Extending analysis to examine the above mentioned issues will be the basis for further research.

In view of the above, the purpose of this study is to determine the diversification of agricultural production and changes in food self-sufficiency, and thus to present similarities in agricultural production levels in the EU candidate countries and the Eastern Partnership countries.

The rest of this article is organized as follows: literature review, materials and methods, results, discussion and conclusions.

## Literature review

A relevant element in the development of agriculture comprises of endogenous factors which determine the economic and social structure from within and result from the natural market processes. The most fundamental are the resources of basic production factors, i.e., land, fixed and financial capital, natural resources and human capital, the internal structure of agribusiness, i.e., the share of individual aggregates in the value of global production or in the total number of employees, the level of technical and economic efficiency of individual entities, expressing the efficiency of resource involvement, the quantity and quality of elements of the technical and social infrastructure of rural areas, the quality of social capital shaping institutional and interpersonal relations in the management processes, individual entrepreneurship and risk propensity among rural residents [29–34]. The said elements crucially influence the development rate of the individual countries’ agricultural sectors, as well as enable to assess the diversification of agricultural production between them.

According to Forteza et al. [35], the agricultural production is a complex socio-economic category, including the resource potential, the production process and the final product, which also implies a level of socio-economic effectiveness. To implement production, achieve the planned goals and solve the assigned tasks, the country must have necessary resources at its disposal. The key to a buoyant economy is the availability of the necessary amount of resources, taking into account their rational use. Characterizing the resources that are at the country’s disposal, such concepts as “resource base” and “resource potential” are very often used. At present, there is no single approach to the definition of these concepts, and, in general, scientists understand under these terms the totality of all the resources that economic entities possess [36].

The production potential of agriculture is a sum of natural resources, methods of their utilization, natural conditions, workforce, technical means and fundamental economic conditions [37,38]. Barthelemy and David [39], as well as Pawlewicz and Pawlewicz [40], emphasized that the proof of the production capacity of agriculture is the presence of production resources. In turn, the ability to utilize the potential and develop optimum relations between production factors has an influence on the efficiency of the production process.

The size, quality and structure of production resources and their efficient use, apart from the social and economic system and economic policy, are the key factors determining the competitiveness of the specific economy and its sectors. The factors shaping production efficiency include the directions of agricultural production, the intensity of management, relations between prices of respective factors and their actual availability [41–44]. Knowledge on the potential production capacity of agriculture is essential since it makes it possible to determine the directions of the agricultural sector development for a specific country or region.

Agriculture plays a fundamental role in human history. Prior to the Industrial Revolution, agriculture was the major economic activity and enabled the human population to grow. As a result, agricultural growth has been regarded as an essential condition, or even a precondition, for the rise of human civilization. However regardless of the increase in agricultural productivity and production efficiency, a process of diminishing of agriculture as a manufacturing department in the national economy can be observed. The limits of this phenomenon, which can assume a dangerous shape of marginalization, are important. In the interest of both farmers and consumers is not to allow agriculture to be marginalized, as food provides for the basic needs of all people [45]. On the one hand, agriculture remains also a vital and unique instrument for achieving poverty alleviation, especially in rural areas [46,47]. On the other hand, agriculture has significant impacts on industrialization, urbanization, and the long-term evolution of the economy [48,49].

Agriculture is a sector of the economy that also has special links with the natural environment. On the one hand, its efficiency depends on the environmental resources; on the other hand, agricultural production often takes advantage of the natural environment (e.g., degrades soil and water quality and reduces biological diversity), which is detrimental to environmental sustainability [50,51].

Conventional agriculture was a product of the agriculture industrialization process, including output intensification, concentration of production potential, and specialization of agricultural holdings [52]. Agricultural production is a complex process that includes the production of food, feed and fibre. It is characterized by the use of various input elements such as machinery, land, water and energy [53]. Agricultural production has developed through the process of intensification as a response to the demand for food, feed and fibre. Sustainable intensification of agricultural production is a key objective to increase production while reducing environmental damage [5].

When Sicco Mansholt started to formulate a European agricultural policy, his primary concern was to ensure that Europe would never again suffer from shortages of food and farmers would have a decent income. The policies implemented to this end were very successful. There has been no deprivation in Europe since, and the continent has gone from importing food to exporting it. At the same time, these policies had unintended consequences and modern agriculture has in many ways become a victim of its own success. Increased production has come at a high price: pollution, destruction of soil, emission of greenhouse gases, poorer animal welfare and, perhaps most perniciously, the erosion of the feeling in society at large that agriculture is an important part of our society. Many people now equate modern agriculture with large scale, heavy use of chemicals and genetic modification (GMO).

Zegar [54] and Kremen and Miles [55] noted that agriculture is a source of half of the local emissions of greenhouse gases other than CO_2_ in the European Union (EU), and one-third of water intake is utilized for its needs. In addition, Zegar [54] noted that agriculture significantly impacts the climate, accounting for nearly one-third of anthropogenic changes and, to some extent, for the loss of biological diversity. The industrial agriculture model, which became very efficient but generated global negative environmental and social effects, proved to be a particular burden to the natural environment.

It is emphasized that the physical limits of the Earth set the ultimate boundaries for all human economic activity. We all know and agree that the key challenge in the coming decades will be to produce enough safe and nutritious food for future populations without running out of resources or destroying Earth’s ecosystems – in other words, without exhausting the biological and physical resources of the planet [56].

## Materials and methods

The investigation covers both the EU candidate countries: Albania, Bosnia and Herzegovina, Georgia, North Macedonia, Moldova, Montenegro, Serbia, Ukraine and Türkiye and the Eastern Partnership countries: Armenia, Azerbaijan and Belarus, as in recent years there has been a shift of countries from the Eastern Partnership group (signed in 2009) to the group of EU candidate countries, and the analyzed countries became candidate countries in the following order: Türkiye (since 1999), North Macedonia (since 2005), Montenegro (since 2010), Serbia (since 2012), Albania (since 2014), Bosnia and Herzegovina, Moldova and Ukraine (since 2022) and Georgia (since 2023). The time frame of the analysis covers the years 2011–2021, as at the time the research and data collection began until that year, it was possible to obtain reliable data from one common database. The following variables were selected for analysis: cereals, fruits, vegetables, milk, poultry and beef, while oilseeds, potatoes and pig production were omitted because this research focused on the analysis of agricultural products, the production of which is particularly important and significant for most of the countries analysed. In order to obtain greater accuracy of data, as the analyzed countries differ significantly in size, the study used per capita indicators. The data employed in this study for comparative purposes were obtained from a single FAOSTAT database and the calculations were made using the STATISTICA program, among others. Various statistical methods were used to illustrate the differences in agricultural production, including: the food self-sufficiency index (%), cluster analysis using the Ward’s method, a synthetic measure of the development of agricultural production calculated using both the non-reference and reference methods, and a composite indicator. The valuation with the help of particular instruments of descriptive statistics is the starting point, but it is not fully sufficient. One-dimensional methods which expertise every single indicator separately provide information about the state and the development of these indicators which is very valuable information in terms of the development of a country, but fractional.

It is valuable to use synthetic methods for the country’s production development as it is possible to accomplish complex characteristics. Composite indicators can be easier interpreted than the whole complex of individual variables and enable fast comparison of countries from a given aspect.

Multidimensional data analysis consists of a set of methods used to analyse multidimensional (multi-feature) communities, i.e., groups of individuals characterized by many features at the same time. Linear ordering methods, which constitute the basis of multivariate comparative analysis (WAP), have been widely used. WAP allows one synthetic variable to be built from a set of variables. Bartosiewicz [57] and Młodak [58] emphasize that the basic goal of taxonomic analysis is to assess the level of differentiation of objects described by a set of statistical features and to determine clusters of these objects in terms of similarity of development, as well as to obtain homogeneous classes of objects due to the properties that characterize them. It is worth noting that too large a set of features may disrupt or even block the ability to effectively classify objects. Additionally, one more aspect should be noted. It is preferable to select indicator variables for taxonomic analysis. Remaining with absolute values may lead to distorted results. Additional assumptions must also be made when selecting features. We eliminate from the set features for which the absolute value of the coefficient of variation is below a certain arbitrarily set threshold value. The most common coefficient is 0.1. We also set a certain threshold value for the diagonal elements of the inverse matrix. The most common number is 10.

The cluster analysis used is a data analysis tool whose aim is to arrange objects into groups in such a way that the degree of connection between objects with objects belonging to the same group is as high as possible and with objects from other groups as small as possible. They do not assume any criterion for assigning objects to groups. The only internal division criterion is the mathematically defined similarity between objects; the number of groups (clusters) is usually not specified in advance. It is a method of grouping elements into relatively homogeneous classes. The basis for grouping in most algorithms is the similarity between elements – expressed using the similarity function. Grouping involves isolating groups (classes, subsets). Cluster analysis can be used to detect structures in data without deriving an interpretation/explanation. In short, cluster analysis only detects structures in the data without explaining why they occur.

The most direct way to calculate the distance between objects in a multidimensional space is to calculate the Euclidean distance. If we have a two- or three-dimensional space, this measure determines the actual geometric distance between objects in the space. It is calculated as follows:


$$\text{Distance}\left(\text{x,}y\right)\text{=}{\left\{\;{\left(\text{xi}-\text{yi}\right)}^{\text{2}}\;\right\}}^{\text{1/2}}$$ (1)

It should be noted that Euclidean distances (and squared Euclidean distances) are calculated based on raw data, not standardized data. This method has some advantages. However, distances are greatly influenced by unit differences between the dimensions from which distances are calculated. This may result in completely different cluster analysis results. In general, it is good to standardize so that you have data on a comparable scale. The features cannot be too heavily correlated with one another, as it results in a multitude of information repetitions entered into the system. In theory, the number of all variables may be substantial; however, in order for the analysis to be practically useful, their amount should not be too extensive [59]. The fundamental goal of the multidimensional comparative analysis (MCA) is to organize the set of units according to a specific criterion. Therefore, the aggregate (synthetic) indicator is established on a set of features that directly determine the properties of units from the studied population [60]. Zeliaś [61] notes that too many features may disturb or even obstruct the prospect of effective unit classification.

Młodak [58] notes that the use of different grouping methods leads to various results, with various arrangements of objects belonging to individual clusters in an optimal – from the point of view of a given approach – combination. These discrepancies may be caused by both the properties of measuring the distance between objects and clusters, the setting of the threshold value of lack of similarity, and the hierarchization technique itself. However, Ward’s positional method is the most effective.

For the composite indicator the ratio method can be characterised by the formula below. In the case of robustness, we use the ratio method with a median.


$$y_{ij}=\frac{x_{ij}}{{\tilde{x}}_{\cdot j}}$$ (2)

Note: y is the modified value, index i refers to country, index j to variable; i =  1, …., 6; j =  1, …, m; where m is the number of variables; x_ij_ is the original values of the variable; ${\tilde{x}}_{\cdot j}$ is the median of the variable. The variables *y*_ij_ are as follows: beef, cereal, fruit, milk, poultry and vegetable production. The composite indicator (CI) is then based on the average of *y*_ij_.


$$SI_{i}=\frac{\sum_{j=1}^{m}y_{ij}}{m}$$ (3)

## Results

Since the issue of diversification of agricultural production has strong links with the stage of development of agriculture (area land, use of fertilizer, use of plant protection, etc.). Thus, it does not allow the direct transfer of a situation experienced from one territory to another without considering the local specificities in the analysis because as emphasized earlier many factors influence the amount of agricultural production in a given country, including: the potential of available resources, which is very diverse in the analyzed group of countries, as it depends not only on the diverse natural and climatic conditions of these countries, but also on various historical conditions of development in these countries.

The importance of the agricultural sector is demonstrated for example by its share in the GDP of a given country, the highest in Albania (over 17%) and comparable in Armenia, Ukraine and Moldova (approximately 10%) (Table 1). Employment in the agricultural sector is also important, with the highest levels in Georgia, Moldova, Albania, Azerbaijan and Armenia (around 30–40%). However it is not necessarily the countries with a higher share of agriculture in GDP (or employment) that display better wealth. The production potential of the agricultural sector also depends on the amount of agricultural land owned and its share in the country’s area. Ukraine, Moldova, Armenia and Azerbaijan have a significant share (around 60–70%), and North Macedonia and Türkiye also have a significant share (around 50%). The significant share of UAA in the country’s area also translates into the amount of UAA per capita, the highest of which is obtained in Ukraine and Belarus (over 0.9 ha/capita). Other indicators that illustrate the possibility of developing agricultural production include for instance the share of arable land and permanent meadows and pastures in the agricultural area. The highest share of arable land occurs in Ukraine, Moldova, Serbia and Belarus (about 70–80% of UAA), while the highest share of meadows and pastures occurs in Montenegro and Georgia (from 80–90% share). To present the possibilities of developing agricultural production in a given country, one more indicator was selected from among numerous indicators, namely the share of UAA in the area group above 100 ha, the highest of which was recorded in Ukraine (over 90%) and is equally significant in Moldova and Serbia (over 50%). Also, the above short description of the potential of the agricultural sector of the surveyed countries reveals very large differences, which may also translate into differences in the production per person in the analyzed group of countries. Moreover, it is worth adding that some of these countries (Albania, Montenegro, North Macedonia, Serbia and Türkiye) have been receiving financial resources, among others, from IPARD for many years and the EPC from ENI, which could also have influenced to some extent the differentiation of the level of production potential of the agricultural sector and the volume of agricultural production obtained in these countries.

**Table 1:** Selected elements of production potential of the agricultural sector in CC and EPC in 2021.

	Share in GDP (%)	Share in employment (%)	UAA share in country area (%)	UAA/capita	Share of AL in UAA (%)	Share of meadows and pastures in UAA (%)	UAA in AL over 100 ha (%)
Albania	17.7	34.6	41.5	0.41	52.8	39.5	
Armenia	11.3	30.3	58.8	0.60	26.5	69.9	4.7
Azerbaijan	5.9	34.2	57.8	0.50	43.7	50.6	26.2
Belarus	6.8	8.1	40.3	0.90	68.8	30.0	
Bosnia and Herzegovina	5.0	11.3	44.2	0.67	44.5	50.7	
Georgia	6.1	40.4	34.3	0.60	13.1	81.5	23.6
North Macedonia	8.7	10.8	50.0	0.60	33.1	63.7	
Moldova	10.4	37.6	69.0	0.73	75.2	14.9	58.2
Montenegro	6.3	7.4	19.0	0.41	3.5	94.3	8.4
Serbia	6.5	13.9	41.4	0.48	75.0	19.1	51.1
Türkiye	5.5	17.1	49.5	0.45	52.2	38.4	4.1
Ukraine	10.6	14.7	71.3	0.94	79.7	18.2	94.9

Source: own work based on: FAOSTAT 2023 [62].

Moving on to the analysis of agricultural production of CC and EPC, it is worth noting that their production structure is very diversified and some of them have been very focused on plant production for years, while others have been focused on animal production (Table 2). Countries that clearly stand out with their focus on plant production are primarily Serbia and Ukraine (over 85% in the production structure), and then North Macedonia and Moldova (over 70% share of plant production). This is interesting, because North Macedonia is a mountainous country, which does not favour crop production. In turn, Georgia and Armenia have a slight advantage in animal production (a little over 50%).

**Table 2:** Average structure of agricultural production in CC and EPC in 2011–2021 (%).

	Average structure 2011–2021 (%)	
	Plant production	Animal production
Albania	54.1	45.9
Armenia	48.1	51.9
Azerbaijan	56.9	43.1
Belarus	59.7	40.3
Bosnia and Herzegovina	66.9	33.1
Georgia	47.3	52.7
North Macedonia	74.6	25.4
Moldova	71.8	28.2
Serbia	87.4	15.9
Türkiye	63.4	36.6
Ukraine	85.1	14.9

Source: own work based on: FAOSTAT 2023 [62].

In some countries, agricultural production fluctuated significantly in the years under study. It is important to remember that the production volume is influenced by many different factors, e.g., climate and weather conditions, farm structure and historical conditions.

Looking at the production of cereals per person, one can observe great diversity among CC and EPC. Ukraine has the largest cereal production (over 1900 kg/person) with close to one quarter of the EU-27 total harvested production, and it is among the top five countries of the world in terms of maize production (Table 3). It is also high in Serbia and Moldova (over 1400 kg/person), where in the analysed years it increased the most among the analysed countries (more than twice) (Table 4). According to the data collection survey on the agriculture sector in Moldova, more than half of the agricultural land is primarily used to produce cereals, sunflowers, and vegetables, and farmers have increasingly turned to cereals. In Belarus, grain production per person is only half as high (793 kg/person). The government of Belarus has been encouraging local cereal production by simplifying the structure of cereal production for the past few years, leading to an increase in cereal production [63]. However, because of unfavourable weather for cereal cultivation in Belarus, the short-term trends of cereal production growth and output are particularly unstable. Montenegro on the other hand is the country with the lowest cereal production (approximately 11 kg/person) among the countries studied. Montenegro is gradually improving the production of grains even though the agriculture sector is characterized more by fruit production. The analysis shows that in the years under study, cereal production increased in most countries, with the exceptions of Armenia, Belarus and Türkiye.

**Table 3:** Per capita production of selected agricultural products in CC and EPC in 2021.

	Production (kg/capita)					
	Beef	Cereals	Fruit	Milk	Poultry	Vegetable
Albania	11.1	242.1	304.2	354.9	5.1	352.9
Armenia	20.6	55.6	253.9	241.2	3.0	259.7
Azerbaijan	14.1	315.9	169.5	215.6	12.1	176.2
Belarus	36.4	793.0	57.0	819.2	52.9	190.1
Bosnia and Herzegovina	3.8	428.2	109.4	166.9	19.5	238.5
Georgia	5.5	116.4	156.8	156.5	6.8	40.7
North Macedonia	2.0	267.3	264.3	179.7	0.8	333.0
Moldova	2.5	1518.1	468.3	86.5	16.5	67.9
Montenegro	5.9	11.2	94.2	276.2	5.7	32.9
Serbia	9.6	1406.3	236.4	208.1	15.2	101.7
Türkiye	17.4	375.9	295.4	273.7	27.2	314.3
Ukraine	7.1	1960.4	66.4	200.2	31.6	228.8

Source: own study based on: FAOSTAT 2023 [62].

**Table 4:** Dynamics of selected agricultural products per person in CC and EPC in 2011–2021 (%).

	Beef	Cereals	Fruit	Milk	Poultry	Vegetables
Albania	−21.8	0.1	33.0	−6.5	−13.9	52.5
Armenia	25.1	−62.6	15.1	17.5	52.2	−3.4
Azerbaijan	14.0	24.7	18.9	26.3	57.7	32.9
Belarus	18.6	−3.7	57.3	22.3	71.9	1.2
Bosnia and Herzegovina	−36.9	43.3	19.4	−11.6	59.9	24.1
Georgia	−2.1	9.9	46.9	1.7	115.3	−16.3
North Macedonia	−20.7	1.0	−1.8	−6.6	4.3	14.0
Moldova	−6.4	121.3	61.4	−40.8	42.1	−32.8
Montenegro	−20.2	12.1	−28.2	−12.6	29.4	15.0
Serbia	−9.6	18.0	−1.7	5.6	13.2	−28.8
Türkiye	80.2	−20.8	10.2	34.8	21.9	6.1
Ukraine	−18.7	58.6	−0.5	−17.8	37.5	5.2

Source: own work based on: FAOSTAT 2023 [62].

Due to their access to fertile land that is highly suitable for cultivation, most CC and EPC are extremely dominant in the production of fruits and vegetables, and per capita fruit production increased in most countries analysed over the period considered. Implemented programs have increased the use of accessible arable land for agricultural reasons by making available water-demanding seed varieties that can also be cultivated in sub-mountainous and mountainous places. Moldovia is the country among the CC and EPC countries with the highest fruit production (over 468 kg/person). Moldova’s national economy relies heavily on the fruit-growing industry, which accounts for almost 40% of the total value of agricultural output in the country. Fruit production concentrates on apples, plums, sweet and sour cherries, pears, peaches and nectarines, quinces, apricots, soft fruit, walnuts, and table and technical grapes. High fruit production per person also occurs in Albania, Türkiye, Armenia, North Macedonia and Serbia (approximately 200–300 kg/person). Türkiye is very competitive in fruit production and/or price ratio thanks to its favourable climatic conditions and low labour cost; it is a major producer and net exporter of fruits and vegetables [64]. The lowest fruit production per person occurs in Belarus and Ukraine (about 60 kg/person). However, it is worth noting that during the period under study, it was in Belarus and Moldova that production increased the most (about 60%), and it decreased the most in Montenegro (by almost 30%).

Vegetable production also shows huge differences between the countries. Compared to fruit and cereal production (kg/person), production of vegetables (kg/person) is relatively low among the countries in this region, with Montenegro and Georgia the lowest (less than 33 and 41 kg/person). Albania, North Macedonia and Türkiye are the countries with the highest production of vegetables per person among CC and EPC (over 300 kg). In Albania, this production increased the most in the period examined (by over 50%), while it decreased the most in Moldova and Serbia (by about 30%), even though they are one of the four smallest vegetable producers among CC and EPC. During the period under study, the production of vegetables per person in Belarus increased the least. Vegetable production has grown so little in Belarus because fewer people are working the land. In Belarus, the cabbage, carrot, beet, and onion are the most important vegetable crops. High yields can be produced with little to no upfront expenditure (other than perhaps the cost of greenhouses) and can be maintained or stored without much difficulty. Cucumbers are grown on a small scale, but they constitute a significant crop in the southern parts of Belarus. Grown in greenhouses for export to Russia, cucumbers are a specialty of the region’s growers. The crop’s competitiveness relies in part on access to inexpensive power. Although tomatoes are grown in Belarus, they are less profitable than other crops due to unfavourable climate conditions [63].

The next important output to discuss is milk production (kg/person) considering that agricultural output and primary commodity yields are the primary measures of agricultural productivity’s efficacy. Regarding milk production, it is the smallest in Moldova (86.5 kg/person) and, additionally, it was in this country that it decreased the most (by over 40%) over the years analysed. Milk in this country is produced by smallholder farmers and it contributes significantly to household livelihoods, food security and nutrition, but dairy productivity is constrained by poor quality feed resources, diseases, limited access to markets and services and dairy animal low genetic potential for milk production. These are some of the reasons that might have caused the decline in milk production for Moldova coupled with climatic and limited access to land resources. The highest milk production per person occurs in Belarus (over 819 kg/person). It is worth noting that Türkiye, Ukraine, and Belarus milk production are above the EU-27 average. Meat and milk are the two most important animal products in Belarus. Roughly 40% of all animal output can be attributed to milk production. Regarding milk, Belarus focuses mostly on exports. This suggests that state agricultural strategy prioritizes the expansion of these industries. It is also obvious that government support is a significant factor in the increase of production in this industry. Consequently, the sector is dominated by large enterprises that receive government subsidies [63].

In the light of decreasing production in Albania, Bosnia and Herzegovinian, North Macedonia, Montenegro and Ukraine and increasing Turkish production, Türkiye is projected to become the most significant milk producer of the region in the next few years. Türkiye is the country where production increased the most (by almost 35%) during the period under study and is among the countries that have had great potential for animal production based agriculture since time immemorial. This is largely as a result of its climate and agricultural land size. Regarding cow milk production, the western Balkan region showed a growing tendency due to the significant growth of average milk yield. Cow’s milk accounts for most of the milk delivered to dairies across most of the CC and EPC.

Cattle meat production is predominant in the CC and EPC rather than small ruminant production. The main share of animal production is in smallholder households. Beef production varies greatly among the analysed countries. The highest is recorded in Belarus (over 36 kg/person), although the beef sector in Belarus is not very well developed though the effectiveness of existing herds has increased significantly. In 2003, state policy began to prioritise milk and beef husbandry as an export sector in Belarus and the number of cattle slowly increased, although the majority of these animals are within agricultural enterprises. Countries with self-sufficiency in crop production guarantee the abundance of cheap feeds for animal production. This is evident in countries such as Türkiye where production increased by over 80% and Belarus which is significantly beef producers in this region. Due to agricultural land availability, climate and government policies, production is significantly low in North Macedonia and Moldova (only approximately 2 kg/person). North Macedonia is highly meat import dependent with a low sufficiency ratio. During the period under study, this production per person decreased in most countries, exceptions being Armenia, Azerbaijan, Belarus and Türkiye, and it declined the most in Bosnia and Herzegovina (by almost 37%).

Poultry meat production is again the highest in Belarus (almost 53 kg/person) and the lowest in North Macedonia (only 0.8 kg/person); however, it increased in most countries except Albania, and the largest increase was recorded in Georgia (over 2-fold).

After analysing changes in the per capita production of selected products in the field of plant and animal production, it is possible to analyse how the level of self-sufficiency for these products has changed in the examined years (Table 5). The algorithm for calculating the food self-sufficiency indicator is expressed by the following formula: food self-sufficiency =  (domestic production/domestic use) * 100%. To a certain point, the agricultural sector plays an important political role and may become a security issue. However, in economic terms, attaining agricultural self-sufficiency may be a goal only if the productivity is high enough.

**Table 5:** Self-sufficiency of selected agricultural products in CC and EPC in 2011 and 2020.

	Self – sufficiency (%)											
	Beef		Cereals		Fruit		Milk		Poultry		Vegetables	
	2011	2020	2011	2020	2011	2020	2011	2020	2011	2020	2011	2020
Albania	95.3	97.1	60.9	63.7	83.0	89.1	98.2	98.0	41.5	35.0	102.6	109.4
Armenia	81.4	94.4	52.5	31.4	89.8	92.0	99.2	101.4	14.0	26.1	98.7	105.2
Azerbaijan	94.3	104.4	59.2	64.5	110.0	114.6	96.1	96.9	80.9	78.8	97.1	108.7
Belarus	153.6	192.7	102.9	97.3	53.8	68.0	147.3	172.5	112.8	168.9	97.4	108.7
Bosnia and Herzegovina	61.1	33.3	64.3	78.8	77.2	74.7	93.6	93.1	85.2	100.0	92.9	90.7
Georgia	70.0	87.0	36.1	38.7	93.1	115.8	97.5	94.8	22.6	32.9	77.4	70.0
North Macedonia	31.3	28.6	74.6	78.1	121.4	111.5	93.3	83.1	5.7	2.4	146.2	115.9
Moldova	166.7	100.0	98.1	120.1	142.0	158.3	95.7	87.3	71.2	66.7	102.3	82.7
Montenegro	71.4	66.7	12.0	4.9	61.7	58.7	73.8	73.4	44.4	36.4	62.7	58.0
Serbia	102.5	115.5	131.8	159.9	118.9	120.2	103.6	102.2	98.1	92.7	102.9	104.5
Türkiye	83.5	99.8	107.7	95.2	131.1	137.2	100.8	100.9	116.7	130.5	105.6	107.4
Ukraine	102.3	108.5	143.4	404.9	73.1	73.7	105.5	95.8	98.7	129.8	100.0	102.0

Source: own study based on FAOSTAT 2023 [62].

Looking generally at the table on self- sufficiency, it can be observed that during the entire period under study, the highest self-sufficiency rates for almost all analysed agricultural products were recorded in three countries – Serbia, Türkiye and Ukraine – and they have the greatest potential to compete with EU countries. In 2020, Serbia achieved the largest surplus in grain production (almost 60%), Türkiye in fruit production (almost 40%), and Ukraine also had a surplus in grain production (over 4 times). The greatest shortages of basic products in the years under study can be observed in Montenegro and Georgia, and this is most noticeable in the case of cereals and poultry. When it comes to a specific market, in the entire group of surveyed countries the greatest problem with self- sufficiency concerned poultry meat, where the lowest rates were recorded in North Macedonia as well as in Armenia, Georgia and Albania (at around 30%). Some countries also have a clear problem on the grain market, e.g., Montenegro, Georgia and Armenia. In turn, in the beef market, North Macedonia and Bosnia and Herzegovina had very low self-sufficiency (about 30%). A better situation occurred in fruit production, where the lowest self-sufficiency was around 60–70% in Montenegro, Belarus, Ukraine and Bosnia and Herzegovina, and also vegetables, around 60–70% in Montenegro and Georgia. In most countries, the best situation occurred in the examined years on the milk market, where the lowest self-sufficiency occurred again in Montenegro and North Macedonia (approximately 73–83%).

Based on the production of selected agricultural products per person, a cluster analysis was performed for both 2011 and 2021 and the countries were divided into individual groups taking into account the substantive assumptions (Table 6). The decision to divide the clustering tree into single clusters was made based on the cluster analysis performed using the Ward method using the Euclidean distance. Analysis performed for both years under study showed a division into exactly the same four groups of countries. The first group includes Albania, Armenia, North Macedonia and Türkiye, which are distinguished by the highest production of fruit and vegetables per person (on average approximately 280 kg/person and 315 kg/person, respectively), and it is worth noting that in the case of production of these products per person the lowest coefficient of variation values were noted in this group (7.5% and 11.0%, respectively). The second group consists of Moldova, Serbia and Ukraine. These countries had the highest grain production (an average of over 1.6 thousand kg/person) and in the case of this product there was the smallest variation in the study group (less than 15%). The next group comprises Azerbaijan, Bosnia and Herzegovina, Montenegro and Georgia, which had one of the lower production of cereals per person (on average approximately 218 kg/person), fruit (on average 132.5 kg/person) and vegetables (on average approximately 122 kg/person). Belarus was classified separately, with its production per person of all analysed animal products, which stands out compared to other countries, i.e., beef (over 36 kg/person) and poultry meat (almost 53 kg/person) and milk (over 800 kg/person) and at the same time the lowest fruit production (57 kg/person).

**Table 6:** Cluster analysis using the Ward’s method according to the production of selected agricultural products per person in CC and EPC in 2011 and 2021.

Production (kg/person)						
	Beef	Cereals	Fruit	Milk	Poultry	Vegetable
2011						
I group: Albania, Armenia, North Macedonia, Türkiye						
Mean	10.7	282.4	246.7	245.1	7.7	272.2
v (%)	49.7	42.2	9.0	31.8	111.5	9.5
II group: Moldovia, Serbia, Ukraine						
Mean	7.3	1038.0	199.1	195.7	16.0	153.8
v (%)	45.9	24.0	48.1	20.3	31.0	31.3
III group: Azerbaijan, Bosnia and Herzegovina, Georgia, Montenegro						
Mean	7.8	167.0	118.0	207.3	6.9	100.5
v (%)	34.4	69.1	16.9	30.8	51.1	65.4
IV group: Belarus						
	30.7	823.8	36.2	669.7	30.8	187.8
2021						
I group: Albania, Armenia, North Macedonia, Türkiye						
Mean	12.8	235.2	279.4	262.4	9.0	315.0
v (%)	55.5	49.0	7.5	24.1	117.7	11.0
II group: Azerbaijan, Bosnia and Herzegovina, Georgia, Montenegro						
Mean	7.3	217.9	132.5	203.8	11.0	122.1
v (%)	54.5	75.0	23.7	23.3	49.6	72.2
III group: Moldovia, Serbia, Ukraine						
Mean	6.4	1628.2	257.0	164.9	21.1	132.8
v (%)	45.6	14.7	64.1	33.7	35.2	52.1
IV group: Belarus						
	36.4	793.0	57.0	819.2	52.9	190.1

Source: own study based on Table 3.

The method of distinguishing developmental types, which essentially aims at the construction of a synthetic measure, was used to assess the degree of development in the agricultural production of basic agricultural products. Determining the degree of agricultural development is complex, as the change of one indicator results in changes of the others. Occasionally, difficulties occur with regard to the selection of identical indicators for selected research units, as well as the ability to obtain data for selected time periods. In order to assess the level of agricultural production development and therefore the diversity of agricultural production in the analysed countries, the following indicators were used: production per capita of beef, cereals, fruit, milk, poultry and vegetables.

The above-mentioned variables were used for calculations, including both the substantive and statistical assumptions. Having considered the assumption that variables cannot be too heavily correlated with one another, the highest value was obtained on the diagonal inverse of the diagonal matrix 5.4. The indicators were selected in a manner in which their values were characterised by a variable variation, were not excessively correlated and became comparable. The indicators were subsequently changed into stimulants. A stimulant indicates a trait whose high values are desirable and low ones are not. To determine the changes occurring in the diversification of the agricultural production, two methods – pattern and non-pattern – were used.

Table 7 presents the classification of the analysed countries by the level of agricultural production, calculated according to both methods. The measure values for individual countries indicate a strong variation in their level of agricultural production. The application of the above methods allowed for the division the analysed countries into four groups. In the non-pattern method, the values of the obtained measures indicate that in 2011, Belarus and Türkiye comprised the first group with the highest agricultural production, while the average value of the analysed index for these countries in 2011 was around 0.67. Such a high level of agricultural production has been achieved by Belarus due to the highest production per capita of all analysed livestock products such as beef, milk and poultry and by Türkiye the highest production per capita of vegetables. In 2021, only Belarus stayed in group I in the compared periods. Such placement was a consequence of a decrease in cereal production in Türkiye. However, the greatest increase in agricultural production potential occurred in Moldovia, which was promoted from group III (2011) to group II in 2021. The increase of the analysed measure in 2021 in Moldovia resulted mainly from a two-fold increase in the cereal and fruit production per capita. In both years examined, Georgia and Montenegro were in the lowest group. The low level of the analysed index resulted, among other factors, from the lowest production of poultry meat, vegetables, and cereals in Montenegro, and one of the lowest production levels of cereals, milk and vegetables in Georgia compared to other countries. Countries that observed an insignificant decline in the level of agricultural production, but which influenced their position in the compared periods, are Türkiye (from group I in 2011 to group II in 2021) and Armenia (from group II in 2011 to group III in 2021). In Armenia, it resulted from an over 2.5-fold decrease in cereal production per capita and a slight decrease in vegetable production, and in Türkiye from a slight decrease in cereal production per person. The rest of the studied countries in the analysed periods remained almost within the same groups (II, III and IV), which results from very insignificant changes in their agricultural production.

**Table 7:** Level of development of production per capita according to the pattern and non-pattern methods in CC and EPC in 2011 and 2021.

Non-pattern method		Pattern method		Production (kg/capita)					
Group	Country	Group	Country	Beef	Cereals	Fruit	Milk	Poultry	Vegetable
2011									
I	0.737 Belarus	I	0.517 Belarus	30.7	823.8	36.2	669.7	30.8	187.8
	0.608 Türkiye		0.428 Türkiye	9.6	474.5	268.1	203.0	22.3	296.3
II	0.559 Ukraine	II	0.404 Serbia	8.7	1236.1	66.7	243.6	22.9	217.5
	0.558 Serbia		0.384 Ukraine	10.6	1191.8	240.4	197.1	13.5	142.7
	0.498 Albania		0.344 Albania	14.2	241.7	228.8	379.6	5.9	231.4
	0.449 Armenia	III	0.254 Armenia	16.5	148.7	220.5	205.4	1.9	268.8
III	0.430 Moldovia		0.247 Moldovia	2.7	686.0	290.2	146.2	11.6	101.1
	0.420 North Macedonia		0.215 Azerbaijan	2.5	264.6	269.3	192.3	0.8	292.2
	0.347 Bosnia and Herzegovina		0.212 Bosnia and Herzegovina	6.0	298.7	91.7	188.9	12.2	192.3
	0.342 Azerbaijan		0.179 North Macedonia	12.3	253.3	142.5	170.7	7.7	132.6
IV	0.236 Montenegro	IV	0.078 Montenegro	7.4	10.0	131.1	315.9	4.4	28.6
	0.189 Georgia		0.040 Georgia	5.6	105.9	106.8	153.9	3.1	48.6
2021									
I	0.677 Belarus	I	0.731 Belarus	36.4	793.0	57.0	819.2	52.9	190.1
II	0.506 Türkiye		0.692 Türkiye	17.4	375.9	295.4	273.7	27.2	314.3
	0.471 Ukraine	II	0.652 Ukraine	7.1	1960.4	66.4	200.2	31.6	228.8
	0.435 Albania		0.637 Serbia	11.1	242.1	304.2	354.9	5.1	352.9
	0.409 Moldovia		0.631 Albania	2.5	1518.1	468.3	86.5	16.5	67.9
	0.386 Serbia	III	0.611 Armenia	9.6	1406.3	236.4	208.1	15.2	101.7
III	0.370 Armenia		0.608 Moldovia	20.6	55,6	253.9	241.2	3.0	259.7
	0.322 North Macedonia		0.605 Azerbaijan	2.0	267.3	264.3	179.7	0.8	333.0
	0.317 Azerbaijan		0.588 Bosnia and Herzegovina	14.1	315.9	169.5	215.6	12.1	176.2
	0.301 Bosnia and Herzegovina		0.570 North Macedonia	3.8	428.2	109.4	166.9	19.5	238.5
IV	0.163 Georgia	IV	0.521 Georgia	5.5	116.4	156.8	156.5	6.8	40.7
	0.151 Montenegro		0.513 Montenegro	5.9	11.2	94.2	276.2	5.7	32.9

Source: Own study based on Table 3. The countries’ indicators are arranged according to the non-pattern method.

The findings obtained using the pattern method present a similar division of countries in terms of the level of agricultural production as the non-pattern method. The highest agricultural production per capita in 2021 and 2011 occurred in Belarus and Türkiye. Moreover, the applied methods showed that Georgia and Montenegro are the least developed with regard to agricultural production per capita. In the rest of the countries, the level of agricultural production was comparable and they remained within the same groups in the studied periods.

**Fig 1:**
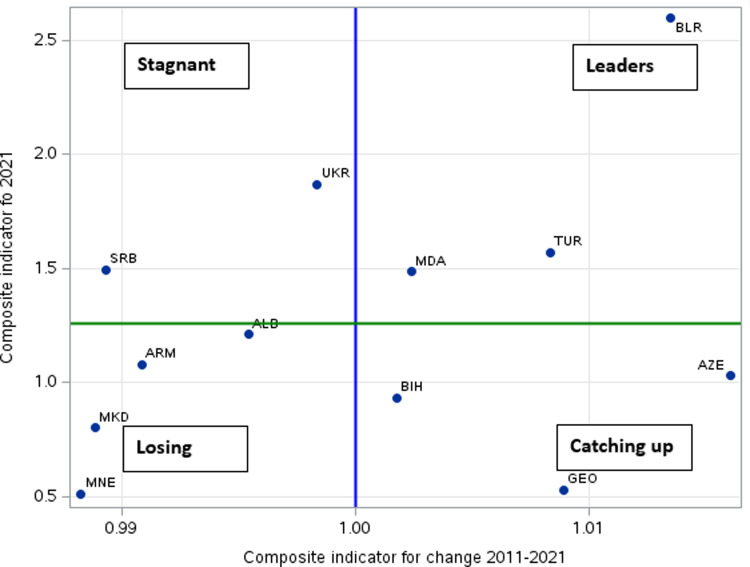
Development diagram for CC and EPC in 2011–2021. Source: study based on Table 8.

The results of the above method are confirmed by the composite indicator, which showed a very similar ranking of countries (Table 8). This method was also used for both 2011 and 2021 to show possible changes in the ranking of the surveyed countries. As can be seen in the figure, the best results in production variables were shown by Belarus both in 2011 and 2021 (Fig 1). Ukraine, Türkiye and Serbia took lower positions, which is consistent with the results obtained from the synthetic measure of production development per capita, as these countries were at the forefront of the analysed countries regardless of the method used; thanks to this, Belarus, Moldova and Türkiye were included in the group of so-called leaders. Next were also placed, very similarly to the previously used method, Albania, Armenia and Azerbaijan, and at the end of the ranking, similarly for both years analysed, Bosnia and Herzegovina, North Macedonia, Georgia and Montenegro. Considering the change from 2011 to 2021, the greatest improvement was shown by Azerbaijan, followed by Belarus and Georgia.

**Table 8:** Composite indicator regarding production in CC and EPC in 2011 and 2021.

	2011		2021		Change 2011 - 2021	
	CI	Rank	CI	Rank	CI	Rank
Albania	1.23	5	1.21	6	0.995	8
Armenia	1.03	7	1.08	7	0.991	9
Azerbaijan	0.90	9	1.03	8	1.016	1
Belarus	2.33	1	2.60	1	1.014	2
Bosnia and Herzegovina	0.91	8	0.93	9	1.002	6
Georgia	0.49	12	0.53	11	1.009	3
North Macedonia	0.88	10	0.80	10	0.989	11
Moldova	1.13	6	1.49	5	1.002	5
Montenegro	0.63	11	0.51	12	0.988	12
Serbia	1.64	3	1.49	4	0.989	10
Türkiye	1.52	4	1.57	3	1.008	4
Ukraine	1.74	2	1.87	2	0.998	7

Source: Own study based on Table 3.

## Discussion

There has been a considerable interest in the phenomenon of production’s diversification, especially during the last two decades [e.g., see references [65–70]]. Summarizing various works that have been undertaken on this subject, it should be noted that the production at the macro level is defined as “the regional volume of output that is possible to produce with the full use of available resources” [37]. In other words, agricultural production can simply be defined as an opportunity to produce a certain volume of output at a certain time.

Agricultural production is one of the most important sectors in many countries. As the global economy becomes more interdependent, countries are concerned with maintaining and enhancing their own production. For EU candidate countries and for EPC with relatively modest economies, agriculture-driven growth is vital. In the framework of the necessary increasing economic convergence for EU membership, enhancing development of production is crucial. Agriculture, despite its intricacy and many irregularities, is vital to the economic development and survival of every nation. It is true that the agriculture sector contributes a small amount to the global economy, but it is nonetheless very important. The economics of agriculture’s growth process have advanced significantly over the past half-century, especially for the EU candidate and the Eastern Partnership countries. Agriculture and rural development can boost the value of non-urban areas’ social income, as measured by rising consumption rates and increases in quality of life.

The importance of agricultural production has increased recently also due to climate change and food security. The exponential growth of the global population increases the demand for food. Although marginal lands including wetland and upland have low productivity, they can be employed to produce food to satisfy the needs of a growing population. In recent years, diseases, weeds, and insect pests have all contributed to a decline in agricultural production while availability of nutrients has a substantial effect on agricultural production and productivity. However, these adverse conditions have not dampened the desire of EU candidate and Eastern Partnership countries to be competitive in regional agriculture production.

In CC and EPC agricultural production is strongly dependent on the structure of the economy and the level of its development. The key areas of efficiency of the agricultural performance for the EU candidate and Eastern Partnership countries are agricultural production and the yields of the main commodities. Only a few CC and EPC are capable of becoming competitive in the EU market for specific agricultural products. The agricultural production of these countries has seen a significant shift in a positive direction over the years, necessitating an assessment of their competitiveness on the EU market. Almost all CC and EPC have implemented new policies and there have been ongoing developments in agriculture that prioritise food safety, environmental protection, and rural development, while also focusing on enhancing market orientation and competitiveness [63,64].

However, it must be added that the EPC have not yet fully exploited their potential benefits [71], which has left them in a weaker comparative position compared to the EU candidate countries. In EPC farming intensity ranges from the relatively intensive farming, through moderate intensity of farming in Belarus, to very low intensity extensive farming in Azerbaijan and Armenia. First two groups of farming intensity are typical for more fertile land with intensive production systems and the third group represents traditional land use, usually found on poorer land. The overall level of yield and productivity is much lower than the EU average due to low application of fertilisers, climate conditions and natural handicaps. Therefore agricultural productivity is quite variable, but there are notable improvements in some sectors and countries (e.g., poultry, milk). Certain countries are still achieving yields at the level of developing countries. Ukraine and Belarus stand apart from the rest in this respect [18].

An anticipated result is that Non-Member States can play an important role in the European markets, primarily due to long-term comparative advantages in production of unprocessed products. To remain competitive, East-European countries will have to change partly their orientation in the agri-food market from commodities towards processing as well as to put more emphasis on export orientation.

The research shows the important role of agriculture in economic development in all analysed countries. For some of them (such as Moldova, and Ukraine) this is one of the most important branches. The manufacturing structure of all the regions is ‘highly specialized’ with a small number of industries having a large combined share in the total manufacturing.

During the last 10 years, the level of specialization in different countries has changed in different directions. In Ukraine, Moldova and Belarus the structure of their economies is not changing so fast and they continue to consider agriculture as one of the main sectors. On one hand, this tendency is quite negative relative to the pace of development of the neighbouring countries. On the other hand, the agricultural specialization despite the war gives Ukraine the chance to become a big player in some segments of the world market (cereal, etc.) and use the current tendencies in the world market for its own development of agriculture and rural areas based on business opportunities (as opposed to the supported agriculture in Europe). Also, in accordance with the regional specialization, peculiarity of location and climate, and national and cultural specific features, most of the countries have some interests in the European and World agri-food market. Their main competitors on the internal European agri-food market are the neighbouring countries, which particularly exert efforts to find new opportunities in the EU agri-food markets. For CC and EPC it is the reference to orient their own agri-food production. Respectively to the concentration of the agricultural production in the region we can also say that country specialization strongly depends on the level of development and economic integration. The example of some countries shows that involvement in the whole European market gives the chance for them to be competitive in agricultural production.

Despite the use of different approaches to regional specialization of agriculture and competitive advantages of CC and EPC, some of the results lead to similar conclusions. First of all, CC and EPC have their own commodities and market shares in the European and World markets and have very favourable conditions to improve their position. There were noted some positive trends in agricultural specialization of some of them relatively to their main competitors. As regards long-term trends in comparative advantage, some countries (such as Moldova and Ukraine) will have a greater advantage in the production of unprocessed products (cereals). Regional specialization of the agriculture of these countries heavily depends on the level of employment (in some states more than 30% of capable people employed in agriculture) and on the historical trends (in some economies agriculture is the main industry) [20], which gives them a greater potential to become a competitive partner in the production of certain agricultural products on the EU market in the future.

## Conclusions

1. The purpose of this study was to determine the diversification of agricultural production and changes in food self-sufficiency, and thus to present similarities in agricultural production levels in the EU candidate countries and the Eastern Partnership countries. It can be said that among the analysed group of countries, we can distinguish in terms of agricultural production Belarus and Türkiye which have differentiated themselves among the researched countries as the dominant ones thanks to large resources of production factors, mainly land, and Belarus thanks to a favourable agrarian structure of farms as well. Due to the high production volumes, Belarus and Türkiye are the states that dominate the agricultural market in the region and have a significant potential to achieve a competitive advantage on the EU market. Albania, Moldova, Serbia and Ukraine display the potential to become competitive countries as well.
2. Due to agricultural land availability, good climatic conditions and government policies Belarus has the opportunity to be competitive on the market mainly of animal products. In turn, three countries dominate the grain market: Moldova, Ukraine and Serbia. It is also worth remembering that during the entire period under study, the highest self-sufficiency rates for almost all analysed agricultural products were recorded exactly in Belarus, Serbia, Türkiye and Ukraine and they have the greatest potential to compete with EU countries. With similar endowments Albania, Armenia and Moldova were able to achieve higher production in vegetable and fruit. In the remaining countries, the possibility of achieving competitiveness on the agri-food market is, for the most part, much more difficult.
3. Unfavourable weather conditions or insufficient agricultural land (mountainous terrain) largely limits agricultural production and place Georgia and Montenegro but also Bosnia and Herzegovina at the bottom of the agricultural production volume per capita. They have a lower production capacity, making it difficult to compete with other nations in the region.
4. To sum up in most CC and EPC the level of development of agriculture and agricultural production is much lower compared to the EU and is quite diversified. This is due to the low level of development of countries, lack of investment in the agricultural sector, access to foreign markets and low productivity, which also results from the historical conditions of the development of these countries. The situation of the agricultural sector and agricultural production was also significantly influenced by numerous economic changes, and above all, the collapse of the USSR and the war in the former Yugoslavia, as well as the agricultural reforms carried out. Therefore, the chances of achieving future competitiveness on the EU market in these countries are often limited.
5. Agricultural production of CC and EPC in the context of future integration with the European Union will probably be subject to a process of some adaptation to the EU, which is perceived as adjustment to the new situation and new conditions, which are, on the one hand, co-determined by the EU Common Agricultural Policy and, on the other, by the state of agriculture in the EU-27 countries, which was also shaped by the CAP. It should be emphasized, however, that this process does not necessarily have to take place by way of adjustment to a specific pattern. Adaptation of agricultural production may therefore mean finding the optimal position possible in the new conditions within the CAP. This adaptation may, over time, affect also the overall cohesion of the agriculture of the EU and thus reduce the differentiation of agricultural production of CC and EPC.
6. Continuing the research issues undertaken, further research will focus on the analysis of the impact of the diversification of the production of agricultural products of CC and EPC on the competitiveness of their foreign trade in agricultural products. In this vein, trade agreements will be analyzed, which play a fundamental role in shaping competition in the agricultural sector. Therefore, it will be examined what happened after the Trade Agreements signed by the Candidate Countries or when the Eastern Partnership was adopted, which may reveal some interesting patterns.

## Abbreviations

CC: candidate countries
CAP: the common agricultural policy
EPC: the Eastern Partnership countries
ENI: The European Neighbourhood Instrument
IPARD: The Instrument for Pre-Accession Assistance for Rural Development
MCA: the multidimensional comparative analysis
SMD: the synthetic measure of development.
